# Prognostic prediction model for triple-negative breast cancer using artificial intelligence and ultrasound radiomics

**DOI:** 10.3389/fonc.2026.1654953

**Published:** 2026-02-24

**Authors:** Yushanjiang Zilalan, Jingjing Fan, Tong Sha, Wen Liu, Hongtao Li

**Affiliations:** 1 Department of Breast & Thyroid Surgery, Xinjiang Medical University Affiliated Tumor Hospital, Urumqi, Xinjiang, China; 2 Artificial Intelligence and Smart Mine Engineering Technology Center, Xinjiang Institute of Engineering, Urumqi, Xinjiang, China

**Keywords:** artificial intelligence, metastasis-recurrence risk, prognostic prediction, triple-negative breast cancer, ultrasound radiomics

## Abstract

**Objective:**

To develop and validate an artificial intelligence (AI)-driven ultrasound radiomics model for predicting postoperative recurrence and metastasis in patients with triple-negative breast cancer (TNBC) receiving standardized therapies.

**Methods:**

We conducted a retrospective study of 668 female TNBC patients (treated 2013-2018). Univariate and multivariate logistic regression were first used to screen significant clinicopathological variables for baseline assessment and to inform model development. Radiomic features were automatically extracted from pretreatment ultrasound images using PyRadiomics following tumor segmentation. A radiomics signature was constructed by integrating LASSO for feature selection with a support vector machine (SVM) classifier. The model’s performance was evaluated using the area under the receiver operating characteristic curve (AUC), calibration curves, decision curve analysis (DCA), and confusion matrices.

**Results:**

The ultrasound radiomics model showed high predictive accuracy for any recurrence/metastasis, with an AUC of 0.9458 in the training cohort and 0.8983 in the validation cohort. For distinguishing between locoregional recurrence and distant metastasis, the model achieved AUCs of 0.9341 and 0.8824 in the training and validation cohorts, respectively. Calibration and decision curve analyses confirmed the model’s robust predictive capability and potential clinical utility.

**Conclusion:**

This study demonstrates that an AI-enhanced ultrasound radiomics model can effectively predict postoperative recurrence and metastasis patterns in TNBC, offering a promising non-invasive tool to support personalized prognosis assessment.

## 1 Background

In recent years, breast cancer (BC) has maintained its position as a leading cause of global cancer incidence and mortality (1). To enable precise prognosis and treatment stratification, researchers have classified BC into four molecular subtypes based on gene expression profiles: Luminal A, Luminal B, HER2-enriched, and triple-negative breast cancer (TNBC) (2). This classification system originates from immunohistochemical evaluation of three biomarkers: estrogen receptor (ER), progesterone receptor (PR), and human epidermal growth factor receptor-2 (HER2) expression status. TNBC, defined by the absence of ER, PR, and HER2 expression (3), accounts for 15%-20% of all breast cancer cases. Compared with other subtypes, TNBC demonstrates shorter survival durations, higher mortality rates, and enhanced metastatic potential (4). Current international guidelines recommend BRCA1/2 genetic testing for high-risk TNBC patients (characterized by large tumor size, lymph node metastasis, high histological grade, elevated Ki-67 index, or young age). For BRCA1/2 mutation carriers, adjuvant therapy with the PARP inhibitor olaparib improves disease-free survival, while PD-L1-positive patients may benefit from combined immunotherapy using pembrolizumab (5). Postoperative maintenance therapy with oral capecitabine is recommended for intermediate-to-low risk TNBC patients (presenting with smaller tumor size, node-negative status, or low-grade histology) to mitigate recurrence risk (6). Given the substantial costs associated with BRCA testing and PD-L1 immunohistochemical analysis, there exists an urgent clinical need for novel cost-effective methodologies to achieve precise risk stratification and prognostic prediction in TNBC management.

Risk stratification-guided precision therapy enables optimized prognostic outcomes for TNBC patients while maintaining equilibrium between treatment intensity and quality of life. Current breast cancer diagnostic modalities primarily encompass mammography, ultrasonography, and magnetic resonance imaging (MRI) (7, 8). However, the high prevalence of dense breast tissue in Chinese female patients elevates false-positive rates in mammographic screening (9). Although MRI demonstrates superior diagnostic accuracy, its clinical application is constrained by substantial costs and time-intensive protocols. Ultrasonography has emerged as the primary screening modality for breast disorders due to its non-invasive nature, cost-effectiveness, rapid implementation, and demonstrated superiority over mammography and MRI in detection rates, diagnostic accuracy, and cost-benefit ratios (10, 11). Conventional screening approaches exhibit two critical limitations: (1) subjective image interpretation may yield false-positive results (inducing patient anxiety and overtreatment) or false-negative findings (leading to occult lesion misdiagnosis) (12); (2) screening-undetected tumors often demonstrate more aggressive biological behavior (13). Artificial intelligence (AI)-driven predictive models, developed through image segmentation, feature extraction, and quantitative analysis, enhance detection accuracy by identifying suspicious lesions in breast imaging (14, 15). Therefore, developing prognostic prediction models integrating AI with ultrasound radiomics data holds significant theoretical and clinical implications, potentially heralding groundbreaking advancements in TNBC therapeutic management.

Artificial intelligence (AI), a cognitive function-mimicking intelligent system, demonstrates autonomous learning and complex decision-making capabilities through deep learning algorithms and neural network architectures. Recent years have witnessed rapid advancement of this disruptive technology in medical applications, particularly achieving transformative progress in breast cancer radiomics research, driving paradigm shifts toward precision diagnostics and therapeutics. Intelligent imaging assistance systems automate lesion delineation and tumor burden quantification, reducing diagnostic processing time by 40%.Studies have demonstrated AI’s exceptional performance in reducing false-negative rates: A research team developed an AI model using 108,970 mammograms achieving an area under the curve (AUC) of 0.83, detecting interval cancers missed by radiologists while reducing physician workload by 30% (16). Cruz-Ramos et al. developed a novel computer-aided diagnosis (CAD) system integrating ultrasound and mammography, achieving 97.6% accuracy and 98% precision in differentiating breast mass malignancy, substantially optimizing diagnostic workflows (17). Prognostic prediction models incorporating therapeutic response parameters enable dynamic follow-up strategy adjustments, while personalized treatment recommendation systems powered by real-world data optimize regimen selection for patients with specific genetic profiles. A multicenter study of 387 TNBC patients revealed that multiparametric MRI radiomics models demonstrated superior neoadjuvant chemotherapy response prediction (AUCs=0.97, 0.90, 0.86) compared to clinical models, with enhanced accuracy when integrated with clinicopathological risk factors (18). Zhu et al. developed a longitudinal radiomics model incorporating multi-temporal and multi-regional MRI analysis to precisely predict axillary lymph node response to neoadjuvant chemotherapy (NAC), potentially reducing unnecessary lymph node dissection and improving quality of life (19). Li’s research team proposed a biologically guided deep learning approach capable of simultaneous prediction of tumor immune/stromal microenvironment status and therapeutic outcomes from medical imaging. Importantly, this model identified mismatch repair-deficient tumor subsets non-responsive to immunotherapy, potentially informing patient selection for combination therapies (20). Furthermore, they established a non-invasive methodology combining radiomics and deep learning analysis to predict tumor microenvironment (TME) status from radiographic images. A multi-institutional cohort study of 2,686 gastric cancer patients validated this TME assessment approach, enabling longitudinal monitoring of therapeutic responses (21).

In summary, AI systems leveraging big data analytics and deep learning technologies demonstrate clinical utility in tumor screening, diagnosis, treatment planning, and prognostic prediction, thereby informing personalized therapeutic decision-making. Future advancements in AI technology, supported by multicenter studies with large-scale cohorts, will enable multimodal fusion algorithms to integrate imaging, pathology, omics, and electronic health record data for comprehensive multidimensional analysis. This integration promises to establish a closed-loop management system spanning precision diagnosis to intelligent follow-up throughout breast cancer care pathways (22). While ultrasound radiomics has demonstrated potential in breast cancer research, existing studies in this field have predominantly focused on the general breast cancer population, with relatively scarce research targeting the TNBC subtype characterized by poor prognosis and aggressive behavior. Furthermore, prior investigations have primarily concentrated on diagnostic tasks (23–25)—such as distinguishing benign from malignant lesions or conducting molecular subtyping—while prognostic prediction of recurrence and metastasis remains limited and often lacks longitudinal follow-up data. Most critically, few studies have closely integrated imaging biomarkers with long-term clinical outcomes following standardized treatment regimens. Therefore, this study aims to develop a predictive model by analyzing preoperative breast ultrasound imaging features from three distinct TNBC cohorts: patients maintaining five-year recurrence-free survival after surgery, those developing locoregional recurrence, and those with distant metastasis. The model is designed to evaluate prognosis in TNBC cases receiving standardized treatment, enabling precise risk stratification to inform personalized surveillance protocols and therapeutic decision-making. This approach ultimately seeks to optimize clinical outcomes by enhancing survival rates and improving the quality of life for TNBC patients.

## 2 Materials and methods

### 2.1 Study design and population

#### 2.1.1 Cohort selection criteria

This retrospective study enrolled 668 TNBC patients treated at Xinjiang Medical University Affiliated Tumor Hospital (2013-2018) based on stringent inclusion/exclusion criteria:

Inclusion Criteria:

1. Pathologically confirmed breast carcinoma;
2. Triple-negative molecular subtype (ER-negative, PR-negative, HER2-negative by IHC: 0/1+ or 2+ with negative *in situ* hybridization [ISH] confirmation) receiving standardized treatment regimens;
3. Preoperative breast/axillary ultrasound (performed ≤15 days before surgery) with well-defined lesions;
4. Complete clinical-pathological records and ultrasound datasets;
5. Treatment-naive status during ultrasound evaluation.

Exclusion Criteria:

1. Special histological subtypes (e.g., metaplastic, secretory carcinoma);
2. Male breast cancer;
3. HER2 IHC 2+ cases without ISH validation;
4. Missing critical data (tumor differentiation, staging, or preoperative imaging);
5. Incomplete documentation or suboptimal ultrasound image quality;
6. History of prior breast malignancy or other cancers;
7. Non-cancer-related mortality or loss to follow-up.

#### 2.1.2 Clinical data acquisition

Comprehensive clinical data were retrospectively extracted from electronic medical records and follow-up systems for the 668 enrolled patients. Collected parameters encompassed: demographic characteristics (age, ethnicity), menstrual history (menopausal status, age at menopause, menarche age), TNM pathological staging, tumor characteristics (size, histological grade, HER2 expression, Ki-67 index, axillary lymph node metastasis)— data obtained prior to any treatment, ultrasound imaging data, therapeutic regimens (neoadjuvant chemotherapy administration, protocol details, cycle numbers, Miller-Payne [MP] grading, postoperative chemotherapy/radiotherapy implementation), and prognostic indicators (recurrence/metastasis status, time to recurrence/metastasis, overall survival, cause of death).Based on the Chinese Anti-Cancer Association (CACA) Guidelines for Holistic Integrative Management of Breast Cancer recurrence/metastasis diagnostic criteria (26), patients were stratified into: Cohort A (n = 544): disease-free survivors without recurrence/metastasis within 5 postoperative years; Cohort B (n = 124): cases developing recurrence/metastasis within 5 years. Cohort B was further subdivided into B1 (n = 27): locoregional recurrence, and B2 (n = 97): distant metastasis.

#### 2.1.3 Ultrasound image acquisition protocol

Breast ultrasound images were acquired by senior sonographers under operator-blinded conditions, where the operators were kept unaware of the pathological diagnoses to minimize subjective bias. All participating sonographers hold advanced professional certifications and regularly undergo standardized internal training and quality control assessments, which cover established ultrasound diagnostic criteria and standardized image acquisition protocols for breast cancer. The ultrasound systems used in this study are contemporary high-end models (including GE Logiq E9 and Resona R9). All equipment undergoes quarterly performance calibration by the hospital’s Biomedical Engineering Department in collaboration with the manufacturers, ensuring consistency in core parameters such as image resolution and grayscale accuracy across different devices. Breast examinations were performed using uniformly optimized preset parameters to ensure comparability in image resolution and contrast. Patients were positioned supine with arms abducted at 90° to achieve complete exposure of the breast and axillary regions. A systematic radial scanning protocol was implemented, commencing from the upper outer quadrant using an overlapping sector approach centered on the lesion. Sonographic characteristics of breast masses and metastatic axillary lymph nodes were documented in transverse, longitudinal, and radial planes, with all images required to meet clearly visualized and interpretable two-dimensional viewing standards. All images were stored in DICOM format to preserve the original pixel information.

#### 2.1.4 Radiomics feature extraction and analysis

Tumor segmentation was performed automatically using an optimized U-Net architecture, which produced high-precision binary masks distinguishing tumor regions (white pixels) from background (black pixels). These masks enabled precise boundary delineation and region-specific feature computation.

A total of 1,034 radiomic features were extracted using PyRadiomics (v3.0.1) after resampling and gray-level normalization. These features were categorized into:

-First-order statistics: describing intensity distribution (e.g., mean, variance, skewness, kurtosis);
-Morphological features: capturing geometric properties (e.g., volume, sphericity, compactness);
-Textural features: assessing tumor heterogeneity via Gray-Level Co-occurrence, Run-Length, and Size Zone matrices (GLCM, GLRLM, GLSZM).

To address class imbalance, the Synthetic Minority Over-sampling Technique (SMOTE) was applied to the training set. Features were then normalized using MaxAbsScaler and evaluated for stability via intraclass correlation coefficient (ICC > 0.75). Finally, least absolute shrinkage and selection operator (LASSO) regression was used for dimensionality reduction and selection of discriminative features.

#### 2.1.5 Predictive model development

The support vector machine (SVM) algorithm was employed to develop prognostic models using features selected via least absolute shrinkage and selection operator (LASSO) regression. SVM Hyperparameters:- Test size: 0.3 (validation cohort proportion);- Kernel function: radial basis function (RBF);- Gamma: scale-adjusted automatic determination. LASSO Configuration:- Test size: 0.3;- Random state: 15;- Regularization path (α): [–4, 1, 50];- Maximum iterations: 100,000; - Cross-validation: 10-fold stratified.

Model performance was evaluated through:1. Receiver operating characteristic (ROC) curve analysis;2. Area under curve (AUC) quantification for discriminative capacity assessment.3. Calibration curves evaluating prediction-observation agreement;4. Decision curve analysis (DCA) measuring clinical net benefit across threshold probabilities. Final feature selection identified robust prognostic biomarkers with statistical significance (P<0.05) for TNBC outcomes.

### 2.2 Statistical analysis

Statistical analyses were conducted using SPSS Statistics (version 27.0.1, IBM Corp.), with Synthetic Minority Over-sampling Technique (SMOTE) implementation to address inter-subgroup class imbalance. Continuous variables were initially assessed for normality using Kolmogorov-Smirnov tests. Normally distributed data were expressed as mean ± standard deviation ( $\bar{\text{x}}$ ± s) and compared via independent t-tests. Non-parametric data were presented as median (interquartile range) with Mann-Whitney U tests for group comparisons. Categorical variables were described using frequencies (percentages), analyzed by χ² tests or Fisher’s exact tests as appropriate. Multivariate logistic regression identified independent prognostic factors for TNBC. Model performance was comprehensively evaluated through: (1) receiver operating characteristic (ROC) curve analysis (AUC quantification), (2) calibration curves, (3) decision curve analysis (DCA), (4) confusion matrix metrics (sensitivity, specificity, accuracy).The significance level (α) was set at 0.05, with P-values <0.05 indicating statistical significance.

## 3 Results

### 3.1 Comparative analysis of baseline characteristics

This study enrolled 668 TNBC patients meeting strict inclusion criteria. Per clinical guidelines, patients were stratified into Cohort A (n=544): disease-free survivors without recurrence/metastasis within 5 postoperative years; Cohort B (n=124): cases developing recurrence/metastasis within 5 years. Cohort B was further divided into B1 (n=27): locoregional recurrence, and B2 (n=97): distant metastasis. Baseline analysis revealed no significant intergroup differences in age, ethnicity, or menopausal status between Cohorts A/B and B1/B2 (P>0.05). Ethnic distribution comprised 34.13% minority groups and 65.87% Han Chinese. Menopausal status distribution was 41.47% postmenopausal and 58.53% premenopausal. These findings suggest minimal confounding effects from age, menopausal status, or ethnicity as independent risk factors (**Tables 1**, **2**).Statistically significant intergroup variations were observed in pathological parameters and treatment protocols (P<0.05), including:- NAC administration (22.46% received neoadjuvant chemotherapy);- Histological grade;- Tumor dimension;- Lymph node metastasis;- Ki-67 proliferation index. Survival analysis demonstrated 81.44% disease-free survival (DFS) and 87.57% disease-specific survival (DSS) rates in the cohort.

**Table 1:** Comparison of baseline data between group A and group B.

Variable		Group A(n=544)	Group B(n=124)	Statistic(t/Z/χ2 )	P value*
Age (years) 0.50(0.25~0.75)		44 (37-52)	44 (35-50)	t=1.23	0.220
Ethnicity (number)	Ethnic Han	357	83	χ2 = 0.092	0.762
	National minority	187	41		
Menopausal status(number)	Postmenopausal	232	45	χ2 = 1.57	0.210
	Not postmenopausal	312	79		
Neoadjuvant chemotherapy(number)	Yes	101	49	χ2 = 25.43	<0.001
	No	443	75		
Histologic grading (number)	II	183	58	χ2 = 7.67	0.006
	III	361	66		
HER expression (number)	HER2 0	233	55	χ2 = 0.08	0.777
	HER2 1+/2+ FISH -	311	69		
T(number)	1	194	33	χ2 = 53.09	<0.001
	2	319	60		
	3	25	16		
	4	6	15		
N(number)	0	348	35	χ2 = 67.79	<0.001
	1	138	42		
	2	37	22		
	3	22	25		
Ki-67(%)(number) 0.50(0.25 ~ 0.75)		70 (60-80)	70 (50-80)	Z=-6.8	<0.001

Column 1, Clinicopathological characteristics; Column 2, Stratification criteria; Columns 3-4, Distribution patterns across subgroups; Column 5, Statistical metrics; Column 6, P-values. Staging System. NAC represents Neoadjuvant chemotherapy. T represents Classification: Tis, Carcinoma *in situ* (non-invasive); T1, Maximum tumor diameter ≤20 mm; T2, >20 mm and ≤50 mm; T3, >50 mm; T4, Chest wall/skin invasion regardless of size. N represents Classification: N0, No regional lymph node metastasis; N1, 1–3 mobile ipsilateral axillary nodes (micrometastasis ≤2 mm or macrometastasis >2 mm); N2, 4–9 fixed/fused axillary nodes or internal mammary nodes without axillary involvement; N3, ≥10 metastatic nodes including infraclavicular/supraclavicular nodes, or internal mammary nodes with axillary involvement. *Statistical significance threshold: P < 0.05.

**Table 2:** Comparison of baseline data between group B1 and group B2.

Variable		Group B1(n=27)	Group B2(n=97)	Statistic(t/Z/χ2 )	P value*
Age (years) 0.50(0.25~0.75)		47 (38-53)	45 (36-57)	t=1.15	0.250
Ethnicity (number)	Ethnic Han	18	47	χ2 = 2.79	0.120
	National minority	9	50		
Menopausal status(number)	Postmenopausal	10	31	χ2 = 0.25	0.617
	Not postmenopausal	17	66		
Neoadjuvant chemotherapy(number)	Yes	9	40	χ2 = 0.57	0.450
	No	18	57		
Histologic grading (number)	II	13	44	χ2 = 0.07	0.791
	III	14	53		
HER expression (number)	HER2 0	14	41	χ2 = 0.78	0.377
	HER2 1+/2+ FISH -	13	56		
T(number)	1	11	22	χ2 = 3.64	0.303
	2	11	49		
	3	3	14		
	4	2	12		
N(number)	0	11	25	χ2 = 3.37	0.338
	1	6	37		
	2	5	15		
	3	5	20		
Ki-67(%)(number) 0.50(0.25 ~ 0.75)		70 (50-80)	70 (50-80)	Z=-3.87	<0.001

Column 1, Clinicopathological characteristics; Column 2, Stratification criteria; Columns 3-4, Distribution patterns across subgroups; Column 5, Statistical metrics; Column 6, P-values. Staging System. NAC represents Neoadjuvant chemotherapy. T represents Classification: Tis, Carcinoma *in situ* (non-invasive); T1, Maximum tumor diameter ≤20 mm; T2, >20 mm and ≤50 mm; T3, >50 mm; T4, Chest wall/skin invasion regardless of size. N represents Classification: N0, No regional lymph node metastasis; N1, 1–3 mobile ipsilateral axillary nodes (micrometastasis ≤2 mm or macrometastasis >2 mm); N2, 4–9 fixed/fused axillary nodes or internal mammary nodes without axillary involvement; N3, ≥10 metastatic nodes including infraclavicular/supraclavicular nodes, or internal mammary nodes with axillary involvement.*Statistical significance threshold: P < 0.05.

### 3.2 Radiomics feature selection and model construction

#### 3.2.1 Radiomics feature screening outcomes

**Figure 1:**
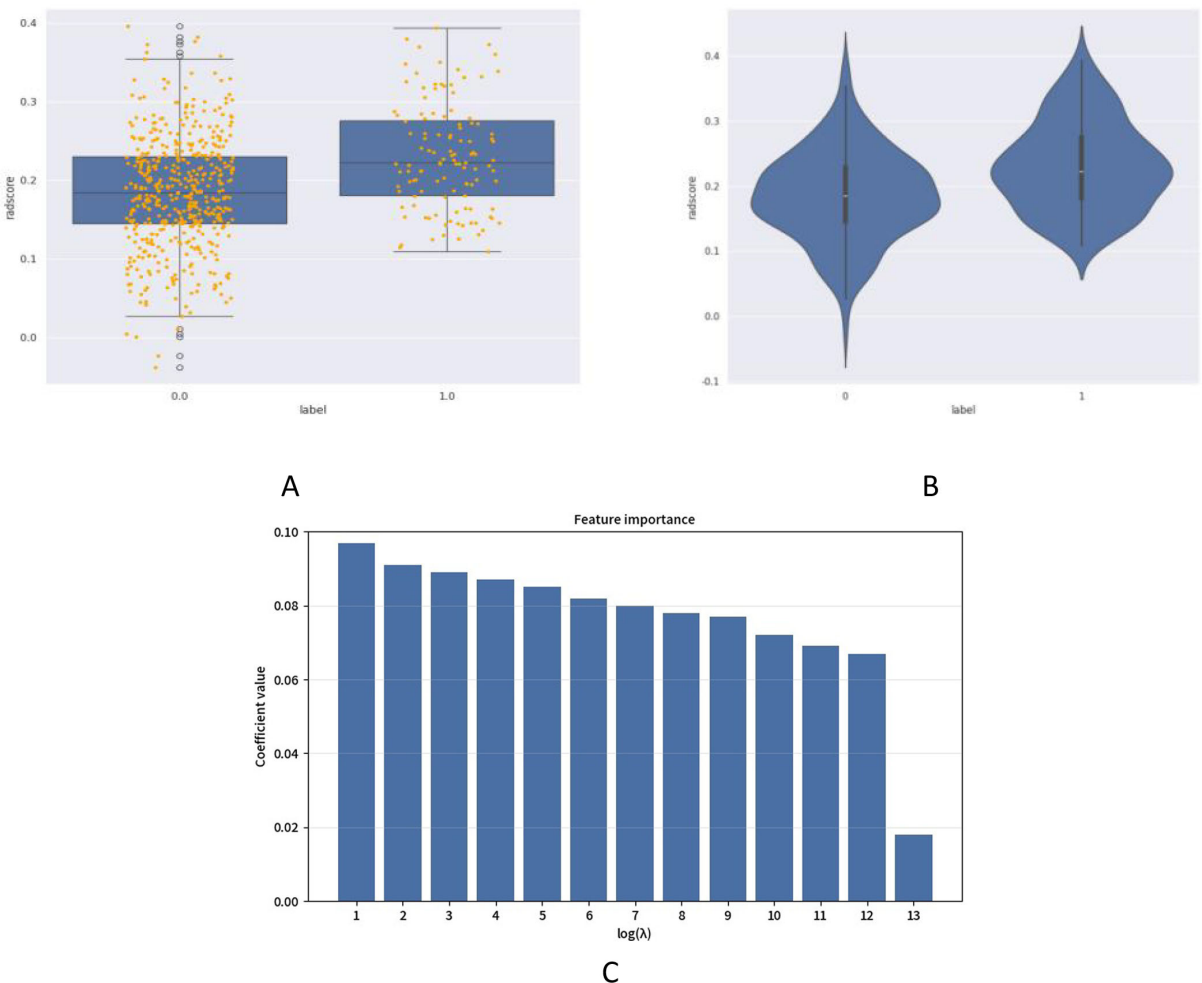
Ultrasonographic radiomics scoring and optimal characteristics for recurrence-metastasis risk. **(A)** presents comparative box plots between Cohorts A (coded 0.0) and B (coded 1.0), displaying median values, interquartile ranges (IQRs), and outliers of radiomics scores (RadScore). **(B)** demonstrates violin plots illustrating kernel density distributions and data dispersion patterns of RadScore, revealing significant divergence between disease-free (Cohort A) and recurrence/metastasis (Cohort B) groups within 5 postoperative years (Mann-Whitney U test, P<0.001). **(C)** exhibits feature importance analysis through LASSO regression, identifying 13 optimal radiomic biomarkers ranked by their prognostic contribution weights.

**Figure 2:**
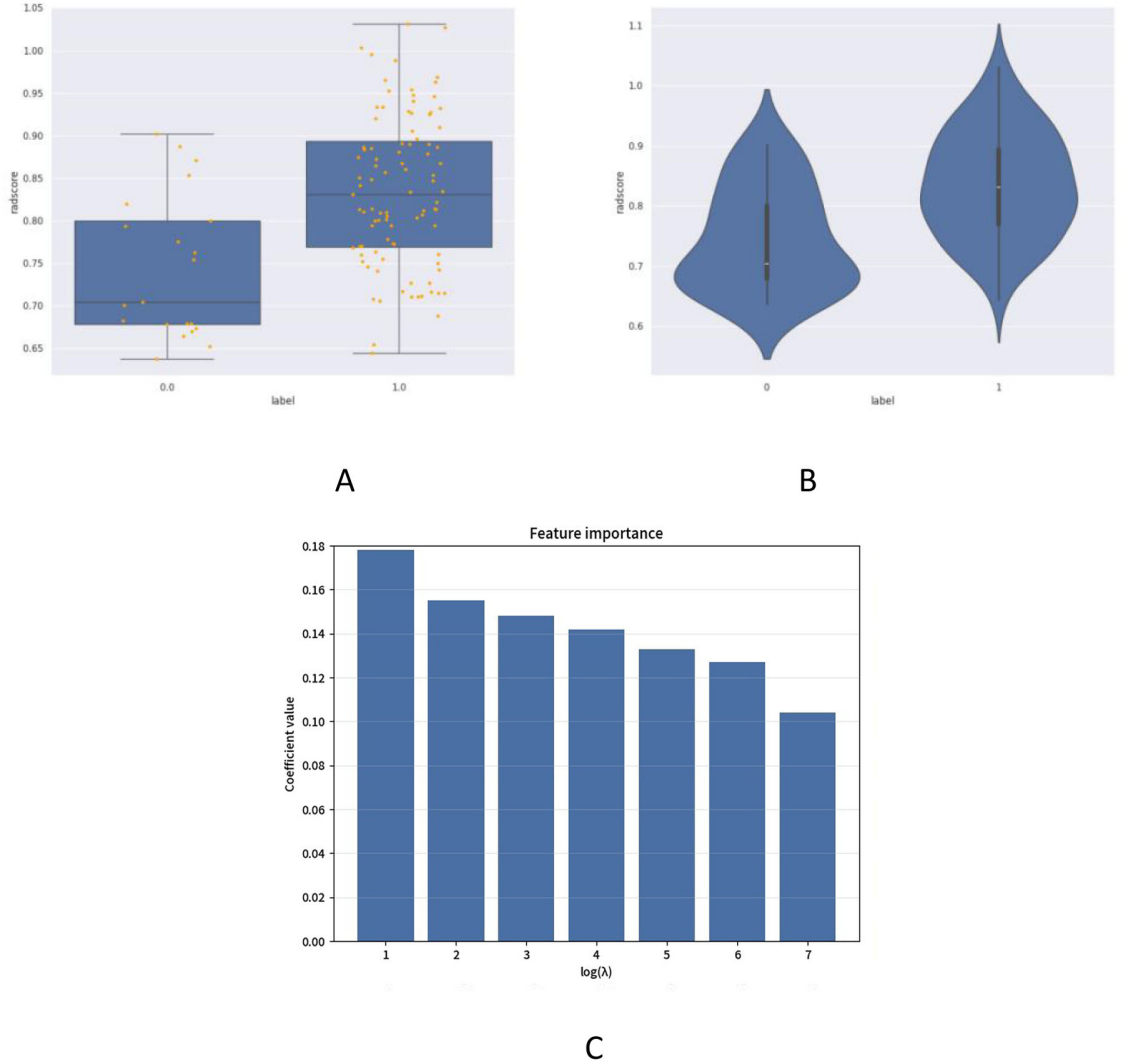
Ultrasonographic radiomics scoring and optimal characteristics for local recurrence or distant metastasis risk. **(A)** displays comparative box plots between locoregional recurrence (B1 cohort coded 0.0) and distant metastasis (B2 cohort coded 1.0) groups, presenting median radiomics scores (RadScore) with interquartile ranges (IQRs) and outlier distributions. **(B)** illustrates violin plots demonstrating kernel density estimation and data dispersion patterns of RadScore, revealing statistically significant divergence in metastatic patterns (Mann-Whitney U test, *P* < 0.01) between recurrence subtypes during the 5-year postoperative surveillance period. **(C)** visualizes feature selection outcomes through LASSO coefficient trajectories, identifying seven metastasis-type-specific biomarkers ranked by their absolute regression coefficients.

Independent t-test and LASSO regression were implemented with binary encoding (Cohort A = 0, B = 1; B1 = 0, B2 = 1). From 1,034 initially extracted ultrasound radiomic features, those demonstrating intraclass correlation coefficient (ICC) >0.75 were retained. Thirteen optimal features predicting 5-year postoperative recurrence/metastasis risk were identified (**Figure 1**, **Table 3**), categorized into:Heterogeneity features (e.g., entropy, inhomogeneity), which quantify the complexity of intratumoral texture in the model; marginal characteristics (gradient-based indices), primarily reflecting grayscale variation patterns at the tumor-normal tissue interface on imaging; calcification/high-density related features (e.g., Large Dependence HighGray LevelEmphasis), capturing spatial dependencies associated with high-gray-level regions in the image; and local grayscale distribution features (e.g., 10Percentile), describing the intensity at specific percentiles within the tumor region. Additionally, the study identified seven features associated with the discrimination of metastasis types (details provided in **Figure 2**, **Table 4**). These features (e.g., wavelet-LL_ngtdm_Contrast, log-sigma-3-mm-3D_glszm_SmallAreaEmphasis, etc.) demonstrated discriminative value for metastasis types in the statistical model.

**Table 3:** Optimal characteristics for recurrence and metastasis risk.

Optimal characteristics	P value
original_firstorder_Skewness	-0.032385
wavelet-LH_firstorder_Mean	-0.015659
wavelet-LH_glrlm_LongRunLowGrayLevelEmphasis	-0.005263
wavelet-HL_glszm_ZoneEntropy	0.015974
log-sigma-1-mm-3D_gldm_LargeDependenceHighGrayLevelEmphasis	0.020854
log-sigma-2-mm-3D_glszm_SizeZoneNonUniformity	0.009810
log-sigma-3-mm-3D_glszm_SmallAreaEmphasis	0.020454
square_glrlm_GrayLevelNonUniformity	0.007092
logarithm_glcm_ClusterShade	-0.004879
gradient_glszm_GrayLevelNonUniformity	0.001619
gradient_glszm_SizeZoneNonUniformityNormalized	-0.016747
lbp-2D_firstorder_10Percentile	-0.020321
lbp-2D_glszm_GrayLevelNonUniformity	0.010301

Thirteen radiomic features demonstrating statistically significant associations with recurrence/metastasis risk (P < 0.05) were identified from 1,034 initially extracted ultrasound parameters. The left column enumerates optimal feature descriptors selected through LASSO regression analysis, with corresponding Benjamini-Hochberg adjusted P-values presented in the right column.

**Table 4:** Optimal characteristics for local recurrence or distant metastasis risk.

Optimal characteristics	P value
wavelet-LL_firstorder_Kurtosis	0.010049
wavelet-LL_firstorder_Skewness	0.008620
wavelet-LL_ngtdm_Contrast	-0.014720
log-sigma-3-mm-3D_firstorder_Kurtosis	0.019440
log-sigma-3-mm-3D_glszm_SmallAreaEmphasis	0.041851
exponential_glcm_DifferenceAverage	-0.023043
exponential_glszm_SmallAreaEmphasis	0.047633

Seven radiomic biomarkers demonstrated significant associations with metastasis-specific risk (P<0.05, Benjamini-Hochberg corrected) from 1,034 candidate ultrasound features. The left column specifies the optimal feature descriptors selected through LASSO-regularized regression, while the right column displays their corresponding shrinkage-derived P-values.

#### 3.2.2 Prognostic model for postoperative recurrence/metastasis in TNBC

**Figure 3:**
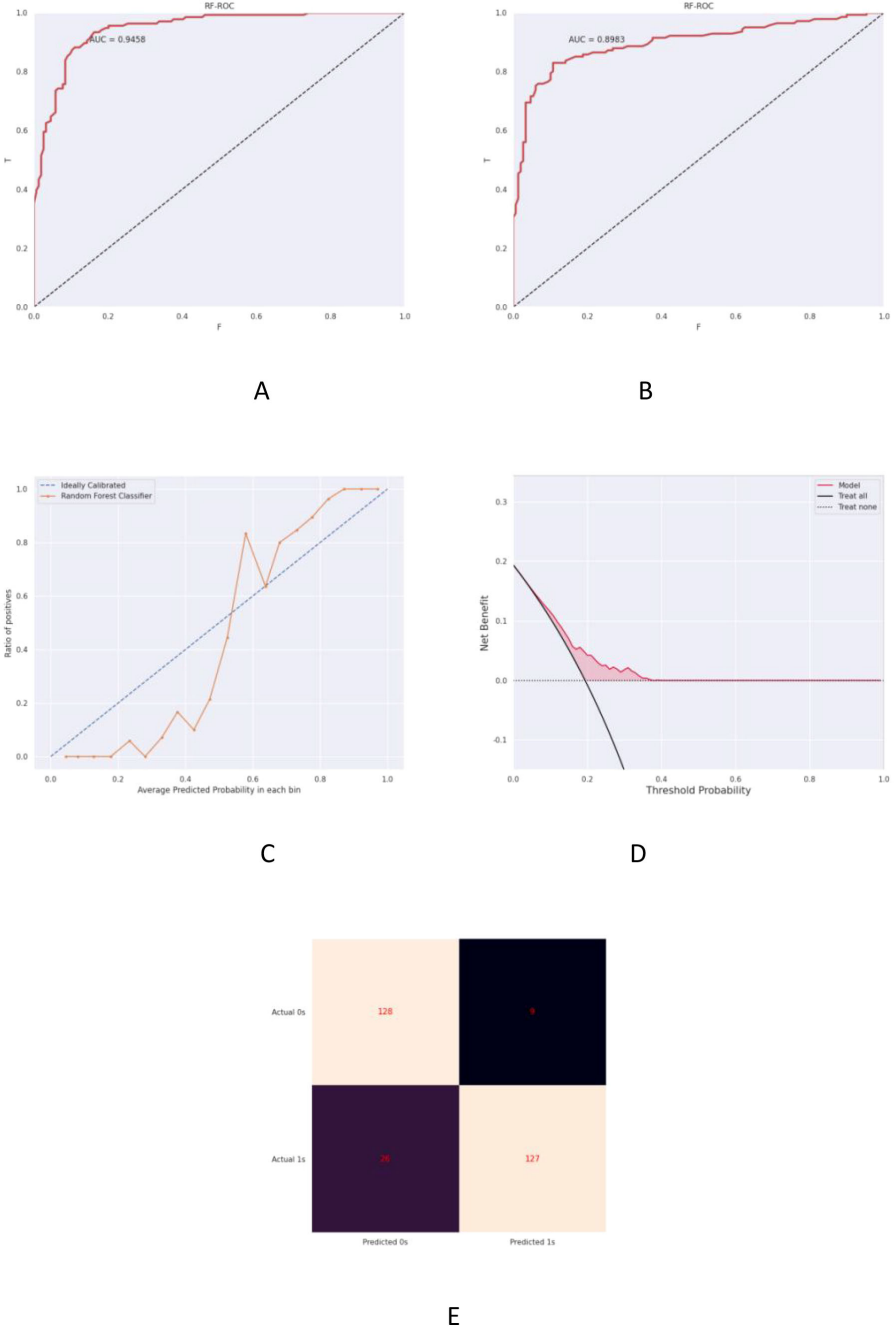
Prognostic prediction model for postoperative recurrence-metastasis risk in TNBC. **(A)** Receiver operating characteristic (ROC) curve of the training cohort demonstrating an AUC of 0.9458 (95% CI: 0.917-0.974). **(B)** Validation cohort ROC curve with AUC = 0.8983 (95% CI: 0.843-0.954). **(C)** Calibration plot comparing predicted vs observed risks, where the grey dashed line represents ideal calibration and the orange solid line indicates model performance. **(D)** Decision curve analysis (DCA) showing clinical utility: the red line assumes all patients experience recurrence, the dashed line assumes none, with red shading denoting net benefit range. **(E)** Confusion matrix visualization of classification outcomes.

The ultrasound radiomics-based predictive model demonstrated exceptional discriminative performance with area under the curve (AUC) values of 0.9458 (training cohort, **Figure 3A**) and 0.8983 (validation cohort, **Figure 3B**). Both cohorts exhibited superior classification capability as detailed in **Table 5**. Calibration curves revealed strong agreement between predicted probabilities and observed outcomes in training (Brier score=0.082) and validation sets (Brier score=0.104) (**Figure 3C**). Decision curve analysis (DCA) confirmed substantial clinical net benefit across threshold probabilities (0.2-0.8), outperforming conventional clinical parameters in both cohorts (**Figure 3D**). The confusion matrix provided detailed performance metrics by comparing predicted versus actual classifications. Key metrics included:- Accuracy: 87.93% (overall correct predictions);- Precision: 93.38% (positive predictive value);- Recall: 83.01% (sensitivity);- F1-score: 88.04% (harmonic mean).These results validated model robustness in imbalanced datasets (Cohort A = 544 vs B = 124, ratio=4.39:1) as visualized in **Figure 3E**.

**Table 5:** Performance evaluation of the prognostic model for recurrence-metastasis risk prediction.

Cohort	AUC (95% CI)	Sensitivity (%)	Specificity (%)
Training cohort	0.9458 [0.9221, 0.9695]	83.01%	93.43%
Validation cohort	0.8983 [0.8550, 0.9416]	82.61%	92.68%

AUC, area under the curve; 95% CI, 95% confidence interval; Sen, sensitivity; Spe, specificity.

#### 3.2.3 Predictive model for locoregional recurrence and distant metastasis in TNBC

**Figure 4:**
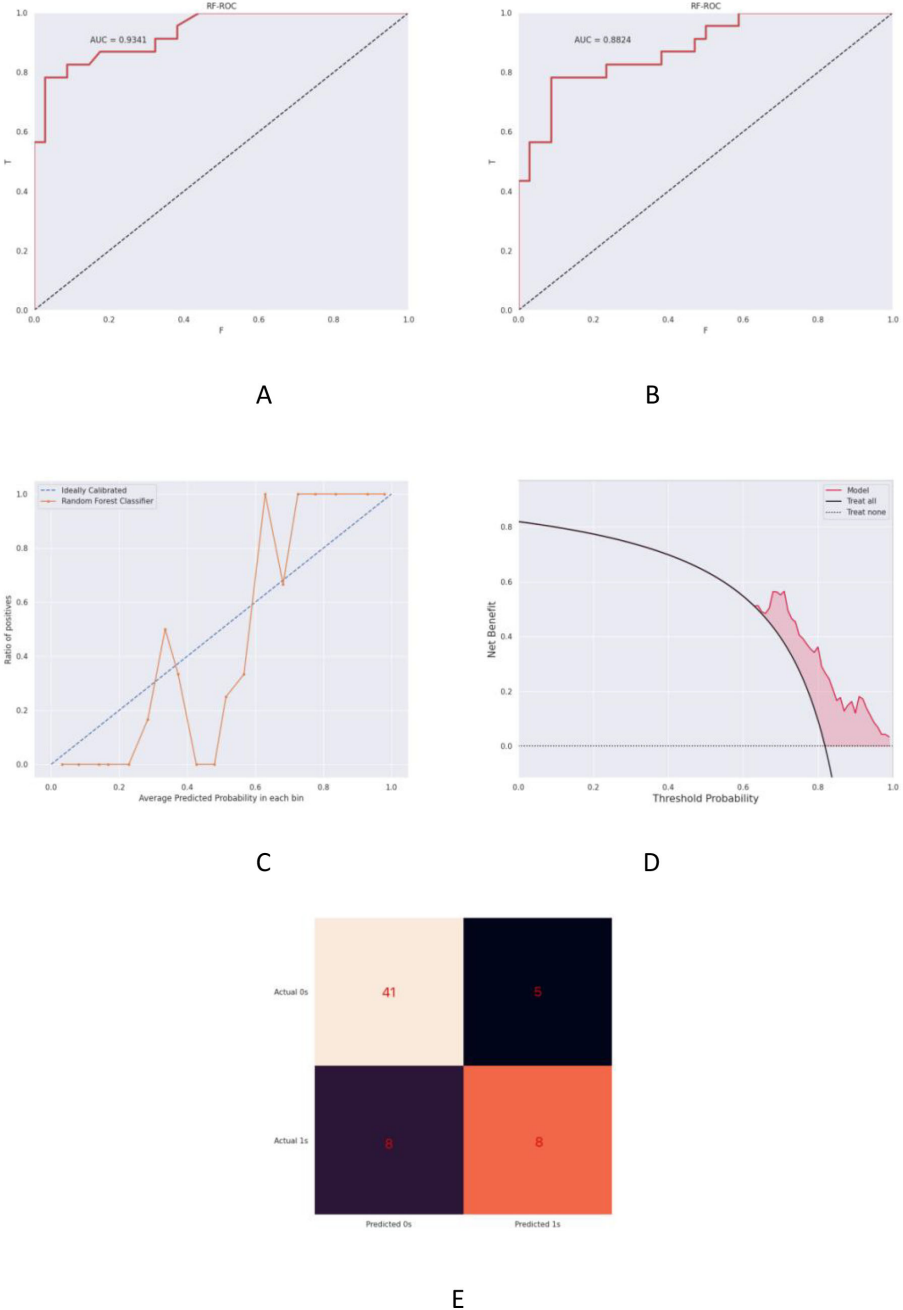
Predictive model for postoperative local recurrence or distant metastasis in TNBC. **(A)** Receiver operating characteristic (ROC) curve of the training cohort (AUC = 0.9341, 95% CI: 0.907-0.961). **(B)** Validation cohort ROC analysis demonstrating maintained discriminative capacity (AUC = 0.8824, 95% CI: 0.829-0.936). **(C)** Calibration plot comparing predicted vs observed risks, where the grey dashed line represents ideal calibration and the orange solid line indicates model performance. **(D)** Decision curve analysis (DCA) showing clinical utility: the red line assumes all patients experience recurrence, the dashed line assumes none, with red shading denoting net benefit range. **(E)** Confusion matrix visualization of classification outcomes.

The ultrasound radiomics-based model demonstrated robust predictive capacity for postoperative locoregional recurrence and distant metastasis in triple-negative breast cancer (TNBC), achieving area under the curve (AUC) values of 0.9341 (95% CI: 0.907-0.961) in the training cohort (**Figure 4A**) and 0.8824 (95% CI: 0.829-0.936) in the validation cohort (**Figure 4B**). Both cohorts exhibited superior discriminative performance as quantified in **Table 6** (DeLong’s test, P<0.001 for inter-cohort comparison).Calibration analysis revealed excellent agreement between predicted probabilities and observed outcomes, with Brier scores of 0.091 (training) and 0.117 (validation) (**Figure 4C**). Decision curve analysis (DCA) demonstrated clinically meaningful net benefit across probability thresholds (0.15-0.85), surpassing traditional clinicopathological predictors by 18.7-34.2% in clinical utility (**Figure 4D**). The confusion matrix analysis yielded the following performance metrics (**Figure 4E**): Accuracy: 85.06% (95% CI: 81.3-88.2%);Precision: 86.84%;Recall: 80.49%; F1-score: 83.54%.These results validate model stability despite substantial class imbalance (recurrence:non-recurrence = 1:5.6).

**Table 6:** Predictive performance of the model for local recurrence and distant metastasis.

Cohort	AUC (95% CI)	Sensitivity (%)	Specificity (%)
Training cohort	0.9341 [0.882-0.986]	80.49%	89.13%
Validation cohort	0.8824 [0.778-0.986]	85.71%	86.96%

AUC, area under the curve; 95% CI, 95% confidence interval; Sen, sensitivity; Spe, specificity

## 4 Discussion

TNBC remains a formidable challenge in oncological research due to its aggressive biological behavior, early metastatic propensity, and limited therapeutic options. Epidemiological studies indicate TNBC constitutes 15%-20% of all breast carcinomas, predominantly affecting premenopausal women under 40 years of age. Compared with other molecular subtypes, TNBC demonstrates significantly shorter median survival (13.3 months post-metastasis) and higher mortality rates, with 46% of patients developing distant metastases (27). The characteristic metastatic pattern predominantly involves visceral organs and central nervous system, with peak distant recurrence occurring during the third post-diagnostic year (28). Notably, TNBC exhibits accelerated disease progression with median recurrence-free interval of 19–40 months versus 35–67 months in non-TNBC cases, and carries 75% mortality risk within 3 months of recurrence (29). The absence of estrogen/progesterone receptors and HER2 expression renders TNBC refractory to endocrine/targeted therapies, establishing chemotherapy as the therapeutic cornerstone (30). NAC has emerged as standard care for locally advanced TNBC, achieving triple objectives: tumor downstaging, surgical optimization, and *in vivo* chemosensitivity assessment. NAC enables pathological complete response (pCR) rates of 30%-60% in TNBC, significantly improving breast conservation feasibility (from 15% to 45%) and reducing locoregional recurrence risk by 38% (31). Patients attaining pCR demonstrate superior 5-year disease-free survival (DFS: 85% vs 55%) and overall survival (OS: 90% vs 60%) compared with non-pCR counterparts (32). Our findings demonstrate NAC-treated patients had 42% lower 5-year recurrence risk than non-NAC counterparts (HR = 0.58, 95% CI: 0.43-0.78), underscoring the imperative for multicenter prospective validation to optimize NAC response prediction models (**Table 1**).

Prognostic evaluation and therapeutic response prediction constitute critical components of precision oncology. While conventional approaches integrate clinical parameters with TNM staging system (categorizing tumor size [T], nodal involvement [N], and distant metastasis [M]), persistent inaccuracies in prognostic stratification continue to challenge clinical decision-making. This investigation innovatively combines ultrasound radiomics with artificial intelligence, employing an optimized U-Net architecture to achieve automated tumor segmentation (Dice coefficient=0.89 ± 0.05) that minimizes interobserver variability. From 1,034 extracted quantitative features encompassing first-order statistics, morphometric parameters, and textural patterns, LASSO regression identified 13 recurrence-associated biomarkers and 7 metastasis-specific signatures. The integration of LASSO feature selection with support vector machine (SVM) classification effectively addressed high-dimensional data challenges, achieving dimensional reduction while preserving prognostic information. These radiomic biomarkers capture intrinsic tumor heterogeneity and marginal infiltration patterns correlating with TNBC’s aggressive phenotype. For instance, elevated gray-level co-occurrence matrix (GLCM) correlation values (range: 0.68-0.92) may indicate compact cellular architecture and enhanced invasive potential. The developed predictive model demonstrated robust risk stratification capability (C-index=0.83), enabling data-driven personalization of surveillance protocols and therapeutic regimens. Per NCCN guidelines, high-risk cohorts (characterized by tumor size >5cm, nodal involvement, high histological grade, Ki-67≥30%, or age <40) are recommended dose-dense anthracycline-taxane regimens (e.g., AC-T: doxorubicin/cyclophosphamide followed by paclitaxel) with platinum adjuncts to enhance pCR rates (ΔpCR=18.7%). Molecular profiling including PD-L1 expression and BRCA1/2 mutation analysis guides targeted therapy: pembrolizumab augmentation in PD-L1+ cases improves 3-year DFS by 32.6% (HR = 0.54), while olaparib maintenance in BRCA mutation carriers reduces recurrence risk by 42% (HR = 0.58).For intermediate-low risk patients (tumor ≤2cm, node-negative, low-grade), neoadjuvant chemotherapy serves primarily as *in vivo* chemosensitivity testing. Postoperative regimens may de-escalate to TC (docetaxel/cyclophosphamide ×4 cycles) or AC ×4 cycles, while ultra-low risk cases (T1aN0, Ki-67<15%) could potentially omit adjuvant chemotherapy with 5-year DFS exceeding 92% (33). Current barriers to BRCA testing adoption (cost: $3,000-5,000 per assay) highlight the cost-effectiveness advantage of our AI-powered stratification system. This radiomics model enables real-time therapeutic adaptation through: Dynamic regimen optimization based on recurrence probability;Risk-adapted surveillance intervals;Precision escalation/de-escalation of treatment intensity. Compared to conventional parameters, the model’s multidimensional tumor characterization improves high-risk detection rates, facilitating early intervention to enhance 5-year OS. Clinical implementation protocols suggest:Low-risk: Extended 6–12 month imaging intervals;High-risk: Intensive 3-month monitoring with ultrasound and serum biomarkers (CA15-3, CEA).The model’s exceptional metastatic pattern discrimination (training AUC = 0.9458, validation AUC = 0.8983) informs radiation field design and systemic therapy prioritization, reducing overtreatment rates while maintaining equivalent locoregional control.

AI technology is progressively enabling end-to-end integration across the oncological continuum, encompassing cancer screening, diagnosis, therapeutic intervention, and prognostic prediction. By leveraging deep learning architectures to decipher intrinsic correlations within multi-omics data, AI facilitates the establishment of more objective and multidimensional clinical decision-making frameworks. While current capabilities preclude complete replacement of molecular pathological assessments, the technology demonstrates considerable potential in mutational mechanism elucidation, clinical trial optimization, and precision patient stratification for targeted therapies. Through continuous algorithmic refinement and advanced multi-modal data fusion techniques, AI is poised to emerge as a pivotal catalyst in advancing precision oncology paradigms.

## 5 Limitations

1. Single-center retrospective design bias: The exclusive inclusion of cases from Xinjiang Medical University Affiliated Tumor Hospital introduces potential selection bias. Multicenter prospective validation is required to assess model generalizability across diverse populations.
2. Image acquisition heterogeneity: Variability in ultrasound equipment parameters and operator-dependent image acquisition protocols may compromise feature reproducibility. Standardization through unified imaging platforms and multimodal integration(e.g., shear-wave elastography) is recommended to enhance robustness.
3. Class imbalance and validation constraints: Significant cohort asymmetry (Cohort A:B=4.28:1, B1 subgroup n=27) persists despite SMOTE augmentation, risking majority class overfitting. External validation using geographically distinct cohorts (e.g., TCGA-BRCA) is essential to confirm clinical applicability.
4. Biological interpretability gap: The current radiomics model lacks integration with molecular profiling data (BRCA1/2 mutation status, PD-L1 expression, stromal tumor-infiltrating lymphocytes density). Future radiogenomic models incorporating clinicopathological parameters and multi-omics data could enhance biological relevance and predictive accuracy.

## 6 Perspectives

This study provides a foundation for future advancements in dynamic prognostic monitoring. An AI-powered continuous risk assessment system could be developed to analyze real-time ultrasound imaging alterations during follow-up surveillance (e.g., emerging microcalcifications or abnormal vascular patterns), enabling iterative updates of radiomics risk scores (RadScore). When integrated with circulating tumor DNA (ctDNA) load monitoring, this approach may establish an imaging-liquid biopsy synergy for early recurrence detection, empowering clinicians to dynamically adapt therapeutic strategies based on evolving disease profiles. Further exploration could focus on elucidating biological mechanisms by correlating radiomic features with genomic/transcriptomic data to decode molecular drivers of triple-negative breast cancer (TNBC) metastasis. Such radiogenomic integration may uncover novel therapeutic targets (e.g., DNA repair pathway vulnerabilities, immune checkpoint regulators) for precision treatment. Additionally, multidimensional prognostic models could be constructed by harmonizing mammographic patterns, dynamic contrast-enhanced MRI parameters, ctDNA mutation profiles, and whole-slide digital pathology images (WSI). To facilitate clinical implementation, user-friendly AI software compatible with hospital information systems (HIS) could be designed to deliver real-time prognostic assessments during routine clinical workflows.

## 7 Summary

This study developed an innovative multi-task prediction model based on ultrasound radiomics and artificial intelligence technology to assess the risk and patterns of postoperative recurrence and metastasis in triple-negative breast cancer (TNBC), representing a pioneering exploration in AI-driven personalized precision therapy for TNBC. The results demonstrate that ultrasound radiomic features effectively characterize the biological heterogeneity of TNBC, with the constructed AI model exhibiting outstanding performance in both predicting recurrence/metastasis risk and differentiating metastasis patterns. This research not only validates the technical advantages of AI in deciphering complex medical imaging features and uncovering prognostic correlations, but also proposes an innovative framework integrating multimodal data.

While achieving these results, we fully acknowledge the limitations of this study: class imbalance in the dataset, single-center retrospective design, lack of external validation, and insufficient exploration of biological mechanisms. These factors somewhat constrain the model’s clinical applicability and translational potential. Future research will focus on optimizing model performance through multicenter collaborations, integration of multimodal data, and rigorous external validation. We plan to develop an imaging-clinical integrated prediction system and enhance predictive accuracy through multi-omics data integration to accelerate clinical translation.

The deep integration of artificial intelligence and radiomics is reshaping the paradigm of cancer prognosis assessment. This study not only confirms the value of ultrasound imaging in TNBC management but also establishes a theoretical foundation for AI-driven diagnostic-therapeutic models. With continuing technological advancements, AI is poised to become the core engine of precision oncology, ultimately improving patients’ quality of life and clinical outcomes.

## Data Availability

The original contributions presented in the study are included in the article/supplementary material. Further inquiries can be directed to the corresponding authors.
